# Vector Transmission Alone Fails to Explain the Potato Yellow Vein Virus Epidemic among Potato Crops in Colombia

**DOI:** 10.3389/fpls.2017.01654

**Published:** 2017-09-25

**Authors:** Diego F. Cuadros, Anngie Hernandez, Maria F. Torres, Diana M. Torres, Adam J. Branscum, Diego F. Rincon

**Affiliations:** ^1^Department of Geography and Geographic Information Science, University of Cincinnati, Cincinnati OH, United States; ^2^Health Geography and Disease Modeling Laboratory, University of Cincinnati, Cincinnati OH, United States; ^3^Corporación Colombiana de Investigación Agropecuaria (Corpoica), Centro de Investigación Tibaitatá Mosquera, Colombia; ^4^Department of Biological Sciences, University of Cincinnati, Cincinnati OH, United States; ^5^Biostatistics Program, Oregon State University, Corvallis OR, United States

**Keywords:** PYVV epidemics, greenhouse whitefly, *Trialeurodes vaporariorum*, vegetative propagules, spatial epidemiology

## Abstract

The potato yellow vein disease, caused by the potato yellow vein virus (PYVV), is a limiting potato disease in northern South America. The virus can be transmitted either by the greenhouse whitefly (GWF), *Trialeurodes vaporariorum* (Westwood) (Hemiptera: Aleyrodidae), or through vegetative propagules, such as infected tubers. Recently, GWF populations have been spotlighted as one of the main drivers of PYVV re-emergence, and consequently, PYVV management has been predominantly directed toward vector control, which is heavily based on insecticide use. However, the drivers of the PYVV outbreaks as well as the contribution of GWF populations on the spread of PYVV among potato crops are still not completely understood. This study aims to assess the role of the GWF as a driver of the PYVV epidemic in the potato-producing areas in Colombia, one of the countries more severely affected by the PYVV epidemic, and whose geography allows the study of the spatial association between the vector and the disease epidemic across a wide altitude range. The geographical clusters where the PYVV epidemic is concentrated, as well as those of farms affected by the GWF were identified using a novel spatial epidemiology approach. The influence of altitude range on the association between PYVV and *T. vaporarioum* was also assessed. We found a relatively poor spatial association between PYVV epidemic and the presence of the GWF, especially at altitudes above 3,000 m above mean sea level. Furthermore, GWF populations could only explain a small fraction of the extent of the PYVV epidemic in Colombia. Movement of infected seed tubers might be the main mechanism of dispersion, and could be a key driver for the PYVV infection among potato crops. Agricultural policies focused on improving quality of seed tubers and their appropriate distribution could be the most efficient control intervention against PYVV dispersion.

## Introduction

Potato yellow vein disease (PYVD), caused by the potato yellow vein virus (PYVV), was first reported in Colombia in 1943 (Salazar et al., 2000). This virus is classified as a *Crinivirus*, a genus that includes the whitefly transmitted members of the family Closteroviridae (Wisler et al., 1998; Salazar et al., 2000). Multiple studies report that PYVV can be transmitted by the greenhouse whitefly (GWF), *Trialeurodes* *vaporariorum* (Westwood) (Hemiptera: Aleyrodidae) (Salazar et al., 2000; Barragan and Guzmán-Barney, 2014), but it can also be spread through vegetative propagules such as infected tubers (Salazar et al., 2000; Sastry, 2013). PYVD symptoms are described as vein yellowing with green interveinal spaces, which are associated with a decrease of photosynthetic capacity, plant vigor, and early senescence (Salazar et al., 2000; Chávez et al., 2009). Affected plants produce significantly less and smaller tubers, reducing yield by 30–50% (Saldarriaga et al., 1988; Tzanetakis et al., 2014). PYVV distribution used to be restricted to Colombia and Ecuador, and had been considered as a re-emergent, local, sporadic epidemic with negligible effects on regional potato yields for more than six decades (Salazar et al., 2000). However, the outbreaks recorded during the last 20 years have reduced significantly potato yields in entire regions of Colombia and Ecuador (Salazar et al., 2000; ICA, 2014), and the virus has been reported throughout potato-producing areas in the Andes region of Venezuela and Peru since 1996 (Salazar et al., 2000; Tzanetakis et al., 2014).

Emergent epidemics caused by a variety of criniviruses have followed the increase in whitefly populations over the last two decades throughout the world (Wintermantel, 2004, 2010, 2016; Tzanetakis et al., 2014). As a result, GWF populations have been hypothesized as one of the main drivers of PYVV re-emergence. Therefore, efforts and resources aimed to control the PYVV epidemic have been focused on controlling GWF populations (ICA, 2014; Learmonth, 2014; Tzanetakis et al., 2014). However, the drivers of PYVV outbreaks and the contribution of GWF populations on the spread of PYVV among potato crops are still not completely understood. Whiteflies are rarely a pest of potato crops, and potato is not its preferred host plant (Learmonth, 2014; CIP, 2016; Godfrey and Haviland, 2016). In addition, whiteflies have low migration rates and reduced inter-crop mobility (van Roermund et al., 1997; Brown and Czosnek, 2002; Whitfield et al., 2015). Furthermore, the presence of the virus has been reported in crops located over 3,000 meters above mean sea level (MAMSL) (Saldarriaga et al., 1988; Franco-Lara et al., 2013), beyond the typical altitude range of the GWF (Byrne and Bellows, 1991; Brown and Czosnek, 2002; Cardona et al., 2005).

Colombia has been one of the countries more severely affected by the re-emergent PYVV epidemic (Salazar et al., 2000; Franco-Lara et al., 2013). Colombia has a complex geographic landscape composed of five natural regions. The largest region, the Andes mountain region, covers a sizeable portion the country. Potato production systems in Colombia are widespread along the Andes, with potato-producing farms spanning a wide altitude range, from approximately 1,000 to 4,000 MAMSL (DANE, 2002). This geographical feature of the potato production system in Colombia, along with the altitude restriction of the GWF home range, generate a unique environment for a natural experiment to assess the association between the vector GWF and the PYVV epidemic.

Against this background, this study aims to assess the role of GWF as a driver of the PYVV epidemic in the potato-producing areas of Colombia. Using a novel epidemiological approach that incorporates spatial statistical methods and Geographical Information System (GIS) techniques, we aim to identify geographical areas where the PYVV epidemic is concentrated and geographical clusters of farms affected by GWF. In addition, we aim to assess the influence of altitude on the association between PYVV and GWF.

## Materials and Methods

### Field Sampling and Data Collection

Potato-producing areas in Colombia were sampled for 1 year between 2013 and 2014. Potato farms were selected using a stratified three-stage random cluster sampling process. Colombia is divided geographically into 32 departments, and nine departments where potato production occurs were selected in the first stage of sampling. In the second stage, 47 municipalities from selected departments were included in the survey. In the final stage of sampling, 569 potato farms from these municipalities were randomly selected with a sample size proportional to the area planted with potato crops in the selected departments, as established by the National Potato Crop Census of Colombia (DANE, 2002). The survey collected data related to the geographic location and altitude of each potato farm, determined through global positioning system (GPS) coordinates.

To determine the presence of GWF, five sampling stations were established in each of the 569 potato farms. The first station was located at the center of the plot, and the other four stations were placed 20 m from the center and equidistant to each other. Each sampling station contained 10 plants, in which the presence/absence of GWF adults and nymphs was recorded.

To detect the presence of PYVV, a bulk sample made of 100 leaflets from 100 randomly chosen plants from each sampled potato plot was collected. Each bulk sample was divided into five sub-samples and stored at -20°C until processing. Total RNA was extracted after grinding the leaflets with liquid nitrogen, using Trizol^®^ reagent (Invitrogen^®^) according to manufacturer directions. The presence of PYVV was determined by reverse transcriptase polymerase chain reaction (RT-PCR), using primers designed for detection of the coat protein (CP) gene of PYVV (F2/3′) (Rodríguez et al., 2009). Briefly, the first strand of cDNA was synthesized using 50 ng of total RNA, 50 U of MMLV reverse transcriptase (Invitrogen^®^), and 0.5 μM of primer 3′ (Rodríguez et al., 2009). For the PCR, 1.6 μl of cDNA, 10 μM of each primer (F2/3′), 2.5 mM of MgCl_2_, and 1 U of GoTaq DNA polymerase (Promega^®^) were mixed in a final volume of 10 μL. The following program was used for amplification: initial denaturation at 94°C for 3 min, followed by 35 cycles of denaturation at 94°C for 1 min, annealing at 55°C for 1 min and extension at 72°C for 1 min, and a final extension at 72°C for 10 min. PCR products were visualized on 1% agarose gels (Invitrogen^®^) stained with SYBR^®^ safe (Invitrogen^®^), and the presence of a single 759 bp band was reported as a positive result for the presence of PYVV in each sample. A leaf sample of *Solanum phureja* cv. “Criolla Colombia” expressing yellowing symptoms (PYVV positive confirmed by RT-PCR) was used as a positive control, and an *in vitro* potato virus-free leaf sample (obtained by *in vitro* meristem culture) was used as a negative control.

### Spatial Clustering Analysis

Two spatial clustering analyses were conducted. The first analysis identified geographical clusters of potato farms reporting the presence of PYVV and the second analysis identified clusters of farms reporting the presence of GWF. Both analyses were conducted using spatial scan statistics (Kulldorff, 1997), implemented in the SaTScan software version 9.4 (Kulldorff et al., 2005; Kulldorff, 2010). Scan statistics are among the most widely used methods for spatial cluster detection. They have been successfully used to support scientific research in epidemiology (Kulldorff et al., 2006; Ryan et al., 2006; Wand and Ramjee, 2010; Cuadros et al., 2013; Jones et al., 2013; Malleson and Andresen, 2015; Ruiz-Grosso et al., 2016). However, only few studies in plant pathology have implemented this methodology (e.g., Coulston and Riitters, 2003; Porcasi et al., 2006; Bayon et al., 2007).

In general, spatial scan statistical analysis locates areas with higher (or lower) numbers of cases than expected under spatial randomness (i.e., cases are uniformly distributed throughout the region). We used separate spatial scan statistical analyses to locate clusters of potato farms where the occurrence of PYVV and GWF is greater than expected by random chance. Briefly, scan statistical analysis uses a computer-intensive search by traversing the study region with a circular scanning window to identify any locations where cases are clustered in space (i.e., locations where there are more cases inside than outside the circular window, under the assumption spatial randomness) (Kulldorff, 2010). By continually varying the radius and center, the procedure produces a very large number of circular windows and therefore a very large number of locations is tested for clustering. A likelihood ratio test was used to determine the statistical significance against the null hypothesis of spatial randomness. Clusters with *P* < 0.05, calculated through Monte Carlo simulations (using the SaTScan default value of 999 iterations), were classified as statistically significant clusters of farms testing positive for presence of PYVV or farms where the presence of GWF was reported. Relative risks were calculated as the observed prevalence of PYVV (or GWF) divided by the expected prevalence of PYVV (or GWF) assuming spatial randomness, both within the scanning window and outside it.

### Statistical Analysis

The statistical analysis was divided into two phases. First, separate simple linear regression models were fitted to assess the association between altitude and the percentage of farms affected by GWF or PYVV. The slopes from both regressions were compared to identify statistically significant differences between them. For the second phase, three simple logistic regression models were fitted using the dichotomous viral status of the farm (i.e., if the farm was affected by PYVV or not) as the dependent variable and the dichotomous GWF status as the predictor variable. All sampled farms were included in the first logistic regression analysis. In the second analysis, only farms located at an altitude lower than 3,000 MAMSL were included. The last analysis included only farms located at or above 3,000 MAMSL.

To estimate the proportion of PYVV positive farms that could be attributed to the presence of GWF, a population attributable fraction (PAF) was conducted using the following equation (Rockhill et al., 1998),

$$\frac{p_{e}(RR-1)}{p_{e}(RR-1)+1}$$

where p_e_ indicates the proportion of source population exposed to the factor of interest (proportion of farms with GWF), and *RR* is the relative risk of PYVV comparing farms exposed to GWF to farms not exposed to GWF. Statistical analyses were conducted using SAS version 9.3 (SAS, 2006), and all GIS analyses and cartographic displays were performed with ArcGIS version 10.3 (ESRI, 2004).

## Results

### Prevalence of PYVV and GWF in Farms

**FIGURE 1:**
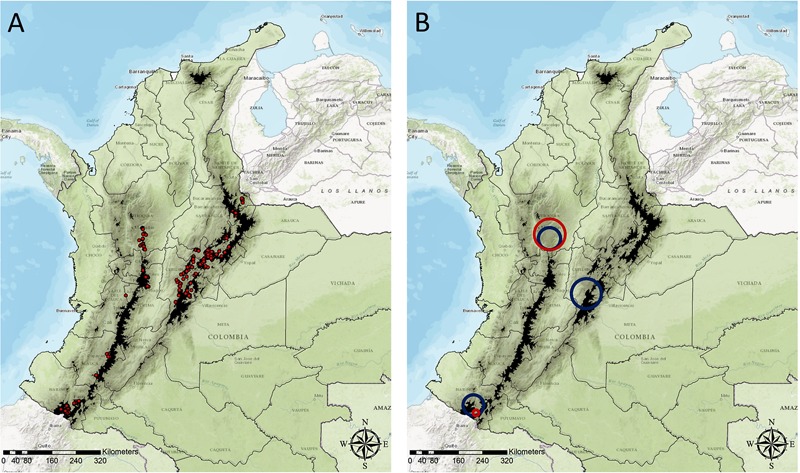
Administrative map of Colombia delineating the different departments, locations of sampled farms, and results of spatial scan statistics analysis. Dark shadowed regions highlight areas above 3000 MAMSL. **(A)** Locations of the 569 farms included in the study. **(B)** Locations of clusters with high numbers of farms where the presence of GWF was reported (red circles), and clusters with high numbers of farms affected by PYVV (blue circles).

A total of 569 farms were included in the sample, from which 229 (39.7%) were located at 3,000 MAMSL or higher (**Figure 1A**). PYVV was detected in 250 (43.9%) farms, while GWF was reported in 131 (23.1%) farms. The simultaneous presence of PYVV and GWF was reported in 98 (17.2%) farms (**Table 1**). Antioquia department was the most severely affected by PYVV and GWF. The majority of farms sampled in this department (71.4%) also reported the co-occurrence of both organisms (**Table 2**).

**Table 1:** Percentage of farms affected by the greenhouse whitefly (GWF) and the potato yellow vein virus (PYVV).

	GWF		PYVV		GWF in PYVV affected farms	
	Presence (%)	Absence (%)	Presence (%)	Absence (%)	Presence (%)	Absence (%)
Total number of farms	131 (23.1)	438 (76.9)	250 (43.9)	319 (56.1)	98 (39.2)	152 (60.8)
Farms at low elevation (<3000 MAMSL)	119 (35.0)	221 (65.0)	179 (52.6)	161 (47.4)	92 (51.4)	87 (48.6)
Farms at high elevation (> = 3000 MAMSL)	12 (5.2)	217 (94.8)	71 (31.0)	158 (69.0)	6 (8.5)	65 (91.5)

**Table 2:** Presence of the greenhouse whitefly (GWF) and the potato yellow vein virus (PYVV) in the nine potato-producing departments in Colombia.

Department	Mean altitude	Total	Number of	Numbers of	Simultaneous
	of sampled	number of	farms affected	farms affected	presence of
	farms (MAMSL)	sampled farms	by PYVV (%)	by GWF (%)	PYVV and GWF (%)
Antioquia	2363	42	36 (85.7%)	36 (85.7%)	30 (71.4%)
Boyacá	2988	224	60 (26.8%)	37 (16.5%)	23 (10.3%)
Caldas	3712	7	0 (0.0%)	0 (0.0%)	0 (0.0%)
Cauca	3082	20	5 (25.0%)	3 (15.0%)	0 (0.0%)
Cundinamarca	2931	161	68 (42.2%)	20 (12.4%)	13 (8.1%)
Nariño	2931	77	68 (88.3%)	29 (37.7%)	28 (36.3%)
Norte de Santander	2565	12	5 (41.7%)	4 (33.3%)	3 (25.0%)
Santander	3248	14	6 (42.9%)	1 (7.1%)	1 (7.1%)
Tolima	3101	12	2 (16.7%)	1 (8.3%)	0 (0.0%)

More than 60% of the sampled farms were located in the departments of Boyacá and Cundinamarca. These departments had low prevalence of GWF, with only 16.5 and 12.4% of the farms reporting the presence of the insect in Boyacá and Cundinamarca, respectively. The presence of PYVV was also low in farms sampled in Boyacá (26.8%), but it was relatively high in farms sampled in Cundinamarca (42.2%) (**Table 2**).

### Spatial Clustering Analysis

Using spatial scan statistics, we identified two geographical clusters with high numbers of farms where GWF was reported, and three clusters with high numbers of farms affected by PYVV (**Table 3**). Although the three clusters identified by the PYVV analysis contained only 26.1% of the total number of farms in the survey, the majority (52.0%) of farms affected by PYVV were in these clusters. Similarly, the two clusters of GWF-affected farms contained 9.4% of the total number of potato farms in the survey, but 36.7% of farms where the presence of whitefly was reported were in these clusters.

**Table 3:** Description of the geographical clusters with high numbers of farms where the presence of the greenhouse whitefly (GWF) was reported, and high numbers of farms affected by and the potato yellow vein virus (PYVV).

Cluster	Radius	*P*-	Number of	Observed	Expected	Relative	Percentage	Percentage
number	(Km)	value	farms inside	number of	Number of	risk	of farms	of presence
			the cluster	farms affected	farms affected		affected (%)	of GWF (%)
*Clusters with high numbers of farms where the presence of GWF was reported*								
1	72.9	<0.001	42	36	10	4.8	85.7	
2	10.3	<0.001	12	12	3	4.7	100.0	
*Clusters with high numbers of farms affected by PYVV*								
1	34.2	<0.001	71	65	31	2.5	91.5	41.4
2	36.5	<0.001	40	36	18	2.2	90.0	85.7
3	49.3	0.01	37	29	16	1.9	78.4	42.1

A cluster of GWF-affected farms and a cluster of PYVV-positive farms overlapped in Antioquia department (**Figure 1B**). These clusters had very high numbers of farms where the presence of GWF was reported (87.1%), and farms affected by PYVV (88.4%). A small cluster of GWF-affected farms was also detected in Nariño department, and this cluster was contained within a much larger cluster of farms affected by PYVV identified in the same area. A third cluster of PYVV-positive farms was located in Cundinamarca department, with PYVV reported in 78.4% of the sampled farms. This cluster did not overlap with a cluster of GWF-affected farms.

### Statistical Analysis

**FIGURE 2:**
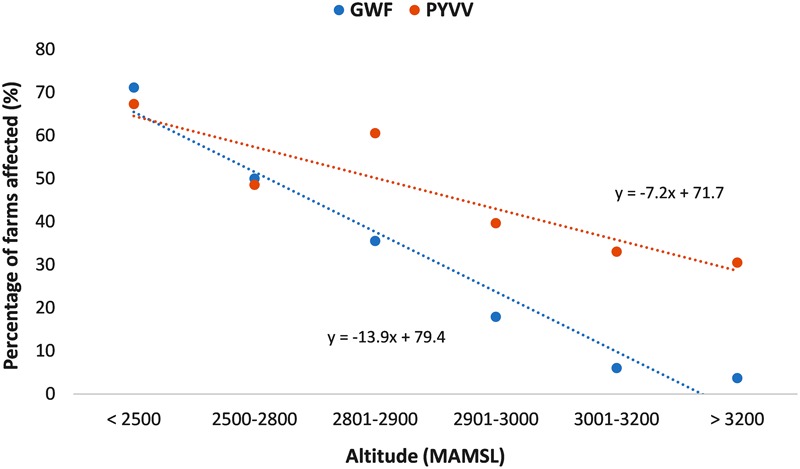
Association between altitude and the percentage of farms affected by the greenhouse whitefly (GWF; blue dots) and the potato yellow vein virus (PYVV; orange dots).

Simple linear regression analysis indicated a statistically significant negative association between altitude and both the percentage of farms affected by PYVV and the percentage of farms affected by GWF (**Figure 2**). However, comparison of the slopes from both regressions indicated a significantly stronger association between altitude and the presence of GWF compared to the association with presence of PYVV (*P* = 0.01).

More than 50% of the farms located below 3,000 MAMSL were affected by both GWF and PYVV. However, GWF was reported in less than 6% of the farms located above 3,000 MAMSL. In contrast, the presence of PYVV persisted at high altitudes, with 31% of the farms located above 3,000 MAMSL testing positive for its presence (**Table 1**).

Farms in which the presence of GWF was reported had almost three times higher odds of being PYVV-positive (Model 1 in **Table 4**; OR = 2.6, 95% confidence interval [CI] 1.9–3.5) compared to farms where the insect was not detected. Similar odds were obtained when including only farms located below 3,000 MAMSL (Model 2 in **Table 4**; OR = 2.7, 95% CI 1.9–3.8). However, the presence of GWF was not significantly associated with PYVV in farms located above 3,000 MAMSL (Model 3 in **Table 4**; OR = 1.4, 95% CI 0.8–2.5). Results from the PAF analysis indicated that GWF populations could be directly responsible for only 27% of the total number of farms affected by PYVV.

**Table 4:** Results from the regression analysis assessing the association between the potato yellow vein virus (PYVV) and the greenhouse whitefly (GWF) presence.

	Odds ratio	95% CI	*P*-value
*Model 1 – Total number of farms*			
Absence of GWF	Ref^∗^		
Presence of GWF	2.6	1.9–3.5	<0.005
*Model 2 – Farms at low elevation (<3000 MAMSL)*			
Absence of GWF	Ref^∗^		
Presence of GWF	2.7	1.9–3.8	<0.005
*Model 3 – Farms at high elevation (> = 3000 MAMSL)*			
Absence of GWF	Ref^∗^		
Presence of GWF	1.4	0.8–2.5	0.24

*The outcome of the regression is PYVV infection.*

*^∗^Reference group.*

## Discussion

According to our results, the PYVV epidemic in Colombia is geographically clustered in three departments: Antioquia, Cundinamarca, and Nariño. The clusters identified in these departments enclosed more than half of the PYVV-positive farms. Still, only a single cluster with a higher number of farms reporting the presence of GWF was found to overlap a cluster of PYVV-positive farms. The overlapping clusters were found in Antioquia, the department where most farms reported the presence of both organisms. Conversely, the largest cluster of PYVV-positive farms identified was located in Cundinamarca, a department that also had the lowest percentage of farms where the presence of the GWF was reported. Potato crops from this department were located at high altitude, at an average of 2,970 MAMSL (Range = 2,171–3,444 MAMSL), compared to crops from Antioquia, which were located at an average altitude of 2,355 MAMSL (Range = 2,130–2,552 MAMSL).

We found that altitude could be an important factor modulating the association between PYVV and GWF populations. Despite the statistically significant association between PYVV and the presence of whitefly reported in Model 1 (all farms included), when only farms located at 3,000 MAMSL or higher were considered (Model 3), this association was no longer significant. Furthermore, there was a strong negative association between altitude and the presence of the GWF, in which the GWF was almost absent above 3,000 MAMSL. Although PYVV was also negatively associated with altitude, the virus was detected in more than 30% of the farms located above 3,000 MAMSL, suggesting that altitude has a stronger negative effect on GWF that on PYVV.

Epidemics caused by viruses such as PYVV could have a more complex natural history than a simple vector-borne transmission. Vector transmission of PYVV might have an important role in virus dispersion, but could fail to explain the extent of the virus epidemic observed in Colombia. In fact, we estimated that the presence of GWF populations could only explain 27% of the total PYVV epidemic in this country. Therefore, other mechanisms of virus dispersion could be driving the PYVV epidemic, particularly at high elevations above the typical range of the GWF distribution.

Viruses such as PYVV have also been reported to be dispersed through vegetative propagules (Salazar et al., 2000; Sastry, 2013), which could be an efficient mechanism of long-distance dispersion boosted by human transportation of infected tubers. According to official data, less than 5% of the area planted with potatoes in Colombia comes from certified seed tubers, and even when seeds are certified, they are not currently tested for PYVV infection (Fedepapa, 2016). Without an adequate seed certification system, informal trade in seed potatoes might provide a key pathway for long-distance virus dissemination, expanding significantly the range of virus dispersion, and becoming a major driver of the epidemic. In addition, it is possible that short-distance dispersion could be stimulated by the distinct yellow color symptoms caused by the virus infection, fueling the ongoing dispersion by attracting GWF populations (Moreau and Isman, 2011). After the establishment of the infection, vector transmission could then become an efficient short-distance mechanism of virus dispersion to other plants within the farm and along the surrounding neighboring crops.

Despite the strengths of our study, a number of limitations are worth noting. First, although a well-developed and validated sampling method was implemented (same within-farm sample size is used for seed tuber certification in several countries), PYVV is commonly distributed irregularly across plants in the field. Therefore, the presence of the virus could have been missed and not reported in some affected farms. Similarly, relying on a reverse-transcriptase PCR protocol for detection of plant pathogens can sometimes lead to misdiagnosis due to very low titer, presence of PCR inhibitors or post-PCR contamination (Wisler et al., 1998; Mumford et al., 2000). However, we tried to minimize these risks in our study, utilizing more reliable primers and an optimized protocol for the amplification of the highly genetically stable coat protein of PYVV, and running a set of positive and negative controls in all the experimental procedures (López et al., 2006). Moreover, this was a cross-sectional study, and temporal changes in both GWF populations and the PYVV epidemic were not captured. Temporal information could provide more insights about the causal relationship between PYVV and GWF as well as their temporal dynamics. Lastly, variables such as tuber origin (i.e., if certified seed was used or not) would have improved our assessment of the association between the PYVV epidemic and viral dispersion through vegetative propagules. Although our survey originally included this variable, we later considered that the information collected on seed tuber origin was unreliable. The rate of potato farmers reporting certified seed tubers estimated was substantially above the official reported rate (<5% official rate vs. 15% reported by farmers in this study). Likewise, current seed tuber certification in Colombia does not include tests for PYVV infection.

Despite these limitations, this is the first study, to our knowledge, that investigates the spatial structure of the PYVV epidemic as well as the association between the virus and its insect vector at a national scale. Outbreaks of GWF populations have been proposed as directly responsible for the recent PYVV re-emergence in Colombia, and efforts have been focused on controlling the insect vector populations. However, without a comprehensive understanding of the epidemic dynamics as well as the main drivers of the viral dispersion, resources to control the epidemic could be mistakenly allocated into interventions that might not be the most effective, and might also cause detrimental economic and environmental impacts. Here, using a large nationally representative sample of potato farms in Colombia, we found that GWF populations can only explain a small fraction of the extent of the PYVV epidemic in the country. Movement of infected vegetative propagules might be the main mechanism of dispersion, and could be a key driver for virus epidemics such as PYVV in agricultural systems. Therefore, designing agricultural policies focused on improving and certifying the quality of tubers and their appropriate distribution could be the most efficient control intervention against virus dispersion.

## Author Contributions

DR and DC conceived the study and its design, conducted the statistical and spatial modeling analyses, and wrote the first draft of the paper. AH and DT contributed to study conception and design, molecular analysis, interpretation of the results, and writing of the manuscript. AB and MT contributed to study conception and design, conduct of the statistical modeling analyses, interpretation of the results, and writing of the manuscript.

## Conflict of Interest Statement

The authors declare that the research was conducted in the absence of any commercial or financial relationships that could be construed as a potential conflict of interest.
